# 1789. Current Antibiotic Prescription Practices in Hospitalized Patients with Acute Myeloid Leukemia (AML) and Febrile Neutropenia of Unknown Origin (FN-FUO): Data from a Large Tertiary Community Hospital

**DOI:** 10.1093/ofid/ofac492.1419

**Published:** 2022-12-15

**Authors:** Carlos Portales-Castillo, Maryrose R Laguio-Vila

**Affiliations:** Rochester General Hospital, Rochester, New York; Rochester Regional Health, Rochester, New York

## Abstract

**Background:**

Optimal duration of empiric antibiotic therapy in hospitalized patients with febrile neutropenia of unknown origin (FN-FUO) remains controversial. Emerging evidence suggests that early discontinuation of antibiotics at 48-72 hours of apyrexia is safe regardless of neutrophil count. However, in the U.S., the adoption of this and other antibiotic use practices in these patients remains unstandardized and understudied, particularly in community hospitals. In this pilot study, we sought to evaluate current antibiotic prescription practices in our large community hospital in admitted patients with AML and FN-FUO.

**Methods:**

We conducted a retrospective cohort study from January 1st, 2020 to December 16^th^, 2021. All patients admitted with a diagnosis of AML and febrile neutropenia (FN) were screened and those with FN-FUO, defined as no microbiological growth on blood, respiratory or urine cultures at 48 hours of first fever were included. Data on initial antibiotic choices, use of vancomycin and its congruence with IDSA guidelines, mean days of therapy (DOT), mean days of “excess” therapy (DOET), defined by days of antibiotics after >72 hours of apyrexia and total DOET were obtained by medical record review and descriptive analysis was performed.

**Results:**

A total of 65 patients with FN were screened. After screening, 27 patients met criteria for inclusion and data from their hospital stay was obtained (Table 1). Cefepime was the preferred choice of initial intravenous β-lactam in 85% of cases and piperacillin-tazobactam was used in the remainder of cases. Vancomycin was used initially in 55% of cases out of which its use was considered to be incongruent with IDSA guidelines in 25% of cases. The most common reason for IDSA incongruent use of vancomycin was lack of documentation for its use. Mean DOT was 10 and mean DOET was 3 (Table 2). In total, we identified 87 excess days of antibiotic therapy in this cohort.

**Figure:**
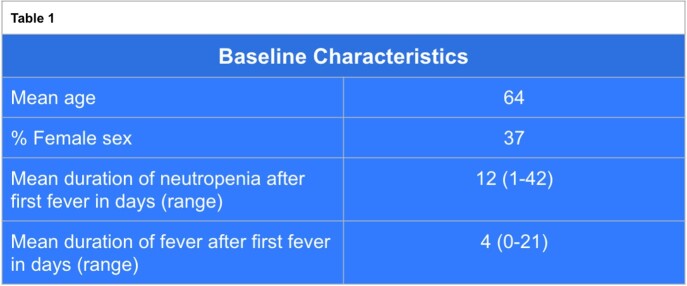

**Figure:**
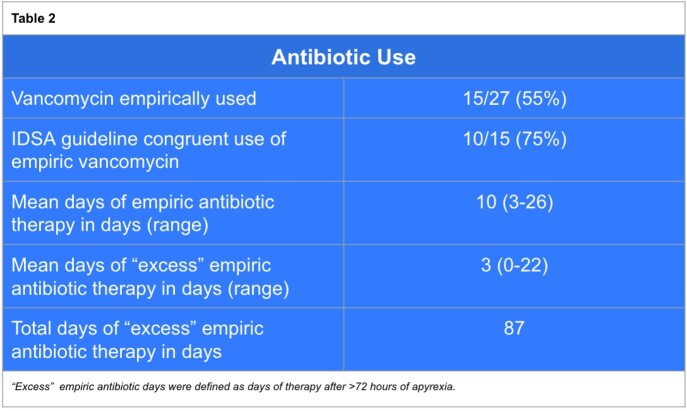

**Conclusion:**

Little is known about antibiotic prescription practices in high risk FN-FUO patients in community hospitals. Our findings highlight potential concrete antibiotic stewardship targets and shortening this knowledge gap may help guide the development of interventions that have been previously shown to improve clinical outcomes in this overall underrepresented population.

**Disclosures:**

**All Authors**: No reported disclosures.

